# *Porphyromonas gingivalis*, a periodontal pathogen, impairs post-infarcted myocardium by inhibiting autophagosome–lysosome fusion

**DOI:** 10.1038/s41368-023-00251-2

**Published:** 2023-09-18

**Authors:** Yuka Shiheido-Watanabe, Yasuhiro Maejima, Shun Nakagama, Qintao Fan, Natsuko Tamura, Tetsuo Sasano

**Affiliations:** Department of Cardiovascular Medicine, Graduate School of Medical and Dental Sciences, Tokyo Medical and Dental University, Tokyo, Japan

**Keywords:** Macroautophagy, Molecular medicine

## Abstract

While several previous studies have indicated the link between periodontal disease (PD) and myocardial infarction (MI), the underlying mechanisms remain unclear. Autophagy, a cellular quality control process that is activated in several diseases, including heart failure, can be suppressed by *Porphyromonas gingivalis* (*P.g*.). However, it is uncertain whether autophagy impairment by periodontal pathogens stimulates the development of cardiac dysfunction after MI. Thus, this study aimed to investigate the relationship between PD and the development of MI while focusing on the role of autophagy. Neonatal rat cardiomyocytes (NRCMs) and MI model mice were inoculated with wild-type *P.g*. or gingipain-deficient *P.g*. to assess the effect of autophagy inhibition by *P.g*. Wild-type *P.g*.-inoculated NRCMs had lower cell viability than those inoculated with gingipain-deficient *P.g*. This study also revealed that gingipains can cleave vesicle-associated membrane protein 8 (VAMP8), a protein involved in lysosomal sensitive factor attachment protein receptors (SNAREs), at the 47th lysine residue, thereby inhibiting autophagy. Wild-type *P.g*.-inoculated MI model mice were more susceptible to cardiac rupture, with lower survival rates and autophagy activity than gingipain-deficient *P.g*.-inoculated MI model mice. After inoculating genetically modified MI model mice (VAMP8-K47A) with wild-type *P.g*., they exhibited significantly increased autophagy activation compared with the MI model mice inoculated with wild-type *P.g*., which suppressed cardiac rupture and enhanced overall survival rates. These findings suggest that gingipains, which are virulence factors of *P.g*., impair the infarcted myocardium by cleaving VAMP8 and disrupting autophagy. This study confirms the strong association between PD and MI and provides new insights into the potential role of autophagy in this relationship.

## Introduction

Cardiovascular disease (CVD) is a major cause of morbidity and mortality, with steadily increasing incidence worldwide. The World Health Organization has reported 17.9 million deaths due to CVDs, accounting for 32% of all global deaths.

Among CVDs, coronary heart disease, particularly MI, continues to be a major cause of death despite significant advancements in its treatment. The global prevalence of MI is approximately 3 million individuals, and >1 million deaths per year have been reported in the United States.^1^ MI occurs when the blood flow in the coronary arteries is blocked by plaque or thrombus, resulting in the inadequate supply of nutrients and oxygen to the myocardium and necrosis of the myocardium.

Periodontal disease (PD) involves the impairment of the connective tissues supporting the teeth and results in the loss of alveolar bone, making it the primary cause of tooth loss in adults. It affects > 50% of the adult population^2^ and is the sixth most common disease worldwide.^3^ A substantial amount of evidence suggests that PD is associated with other systemic diseases, including CVD,^4^ rheumatoid arthritis,^5^ and Alzheimer’s disease.^6^ Among >1000 bacterial species found in the oral cavity,^7^ the “red complex” comprising *Porphyromonas gingivalis* (*P.g*.), *Treponema denticola*, and *Tannerella forsythia* has been strongly linked to the development of advanced periodontal lesions.^8^ *P.g*., a key bacterium in the etiology and pathogenesis of PD, has been detected in approximately 86% of subgingival plaque samples from patients with chronic PD.^9^ This gram-negative anaerobe produces several virulence factors, including lipopolysaccharides, fimbriae, and collagenases, which enable it to evade the host defense system and destroy periodontal tissues. In particular, gingipain protease plays a pathogenic role by facilitating the adherence and colonization of *P.g*. in epithelial cells, causing hemagglutination and hemolysis of erythrocytes, promoting degradation of host proteins, thereby exacerbating the inflammatory response and disrupting host tissues.^10^ The pathogenicity of *P.g*. is not limited to affecting periodontal tissues, as it can invade other cell types and the vessel in human coronary arteries.^11^ Furthermore, studies have detected its DNA in thrombi located at the site of occlusion in patients with acute myocardial infarction (MI)^12^ and atheromatous plaques of coronary arteries.^13^

Therefore, we hypothesize that *P.g*. infection plays a critical role in the pathogenesis of MI. However, the mechanisms underlying the effect of periodontal infection on the pathogenesis of MI and its complications remain unknown.

Autophagy is a critical intracellular process that involves degradation and recycling of cellular components such as proteins and organelles. This process is accomplished by sequestration of these components in double-membrane vesicles called autophagosomes, which then merge with lysosomes for degradation and recycling. Previous studies have shown that dysregulation of autophagy is linked to numerous systemic diseases, including cancer, neurodegenerative diseases, infectious and inflammatory diseases, diabetes,^14^ and CVDs.^15^ Notably, constitutive autophagy in cardiomyocytes is crucial for their survival. Malfunction in the autophagy machinery leads to the accumulation of abnormal proteins and organelles, resulting in cardiac dysfunction.^16^ Therefore, autophagy is crucial for proper functioning of the heart.

Previous studies have shown that *P.g*. invading host cells can escape autophagosomal engulfment and avoid death by autolysosome-mediated bacterial killing. This process is known as xenophagy.^17,18^ We previously showed that invasion of cardiomyocytes by *P.g*. exacerbates post-infarction myocardial fragility and leads to cardiac dysfunction via the suppression of mitochondria-selective autophagy.^19^ Furthermore, we demonstrated that gingipains induce cardiomyocyte apoptosis by activating Bax, a pro-apoptotic protein, through cleavage at its Arg34 site, thereby exacerbating cardiac dysfunction in *P.g*.-inoculated MI mice. However, the molecular mechanism by which gingipains inhibit the autophagy machinery remains unknown. Based on these findings, we propose that *P.g*. interferes with the mechanisms of autophagy and xenophagy, thereby abolishing autophagy-mediated cell protection and exacerbating the pathological condition after MI. In this study, we demonstrated that *P.g*. infection causes dysregulation of autophagy via the inhibition of autophagosome–lysosome fusion, which reduces myocardial viability after MI.

## Results

### *P.g*. invades and worsens the viability of cardiomyocytes by secreting gingipains

**Fig. 1:**
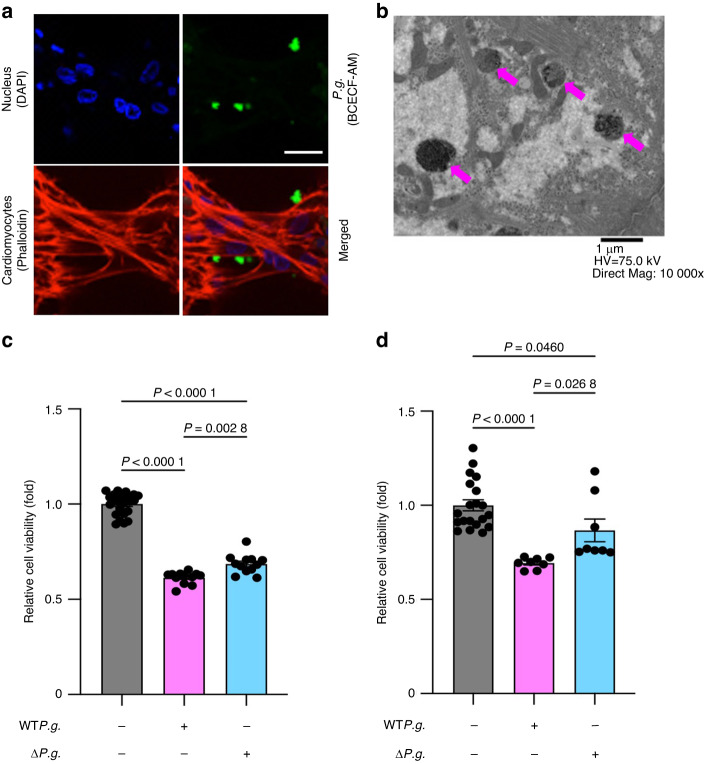
*Porphyromonas gingivalis* (*P.g*.) invades cardiomyocytes. **a**. Fluorescent microscopy images of BCECF-AM–labeled *P.g*. in TRITC-phalloidin–stained neonatal rat cardiomyocytes (NRCMs). Blue, red, and green colors were imparted by 4′,6-diamidino-2-phenylindole, cardiomyocytes, and *P.g*. (scale bar = 25 μm). **b**. Transmission electron microscopic image of *P.g*. labeled with N-acetylmuramic acid through a click reaction in NRCMs. The arrows indicate *P.g*. with gold nanoparticles (scale bar = 1 μm). **c**. The viability of H9c2 cells was assessed among 3 groups, which were treated as indicated (*n* = 12 in each group). **d**. The viability of NRCMs was assessed among 3 groups, which were treated as indicated (*n* = 12 for each group). Results are representative of at least two independent experiments. Data are expressed as mean ± SEM. *P* values were calculated using one-way analysis of variance test followed by Tukey’s post-hoc test for multiple comparisons (**c**, **d**)

*P.g*. invades cells as a survival strategy to evade the host immune system.^20,21^ The presence of *P.g*. within the NRCMs was detected using fluorescence microscopy (Fig.1a), and the presence of *P.g*. within the NRCMs was confirmed using transmission electron microscopy (TEM) (Fig. 1b). Gingipains, which are cysteine proteases secreted by *P.g*., are considered the most potent virulence factors of this organism.^22^ Cell viability assays were performed on both H9c2 cells and NRCMs that were inoculated with either wild-type *P.g*. (WT*P.g*.; strain ATCC33277) or gingipain-deficient *P.g*. (Δ*P.g*.; strain KDP981) to determine the effect of invasion of NRCMs by *P.g*. on cardiomyocytes due to gingipains secretion. The viabilities of H9c2 cells and NRCMs that were inoculated with WT*P.g*. were significantly lower than those of cells or NRCMs that were either untreated or inoculated with Δ*P.g*. (Figs. 1c, d). These results suggest that gingipains produced by *P.g*. has detrimental effects on H9c2 cells and NRCMs.

### Gingipains inhibit autophagosome–lysosome fusion

**Fig. 2:**
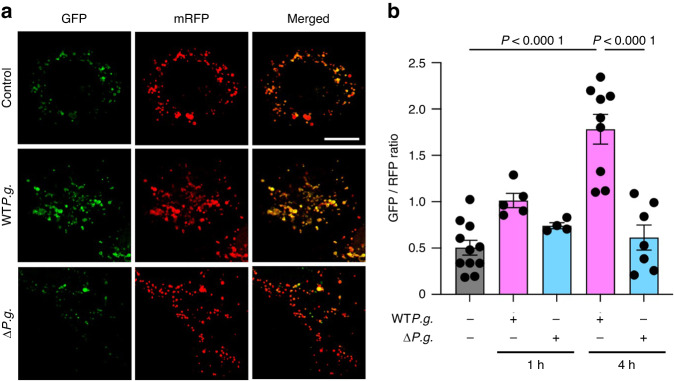
Gingipains from *Porphyromonas gingivalis* (*P.g*.) inhibit autophagosome–lysosome fusion. **a** NRCMs were transduced with adenovirus-harboring mRFP-GFP-LC3 probe and inoculated with *P.g*. for 4 h (scale bar = 10 μm); **b** The graph shows the GFP/RFP fluorescent ratio (*n* = 11 for control, *n* = 5 for 1-h WT*P.g*. inoculation, *n* = 4 for 1-hΔ*P.g*. inoculation, *n* = 9 for 4-h WT*P.g*. inoculation, and *n* = 7 for 4-hΔ*P.g*. inoculation). Results are representative of at least two independent experiments. Data are expressed as mean ± SEM. *P* values were calculated using one-way analysis of variance test followed by Tukey’s post-hoc test for multiple comparisons

Yoshii et al. demonstrated that the formation of autophagosomes in cells expressing mRFP-GFP-LC3 caused an increase in the number of GFP-positive/mRFP-positive (yellow) puncta.^23^ This indicates that a decrease in the number of GFP-negative/mRFP-positive (red) puncta can increase the number of autophagosomes. *P.g*. can evade the host defense system by inhibiting the fusion of autophagosomes and lysosomes.^11^ Gingipains also play a crucial role in allowing *P.g*. to acquire resistance against destruction in the lysosomal system.^24^ Based on these findings, we hypothesized that *P.g*. could suppress the fusion of autophagosomes and lysosomes through a mechanism mediated by gingipains. To validate this theory, NRCMs were transduced with adenovirus-harboring mRFP-GFP-LC3 to monitor the autophagic flux. Confocal microscopy revealed that the GFP/RFP ratio (488/568 nm) in NRCMs inoculated with WT*P.g*. was significantly higher than that in NRCMs inoculated with Δ*P.g*. (Fig. 2a, b), suggesting that gingipains play an important role in *P.g*.-mediated suppression of autophagosome–lysosome fusion.

### Gingipains cleave VAMP8 at its 47th lysine residue site

**Fig. 3:**
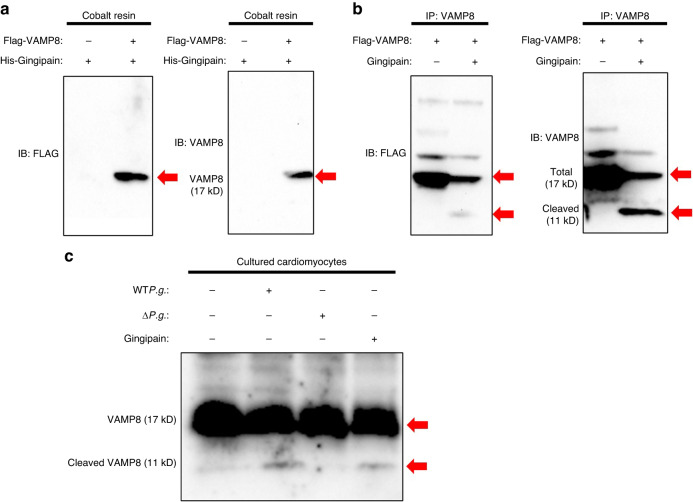
Gingipain from *Porphyromonas gingivalis* (*P.g*.) cleaves vesicle-associated membrane protein 8 (VAMP8). **a** Pull-down assays results indicating physical interactions between gingipains and VAMP8. Recombinant His-tagged gingipain protein was immunoprecipitated with cobalt resin followed by probing with antibodies specific for FLAG and VAMP8. The arrows indicate gingipains physically interacting with VAMP8. **b** Recombinant Flag-tagged VAMP8 protein was precipitated with VAMP8 and incubated with gingipains followed by probing with antibodies specific for FLAG or VAMP8. The gingipains cleave VAMP8 recombinant protein. The arrows indicate VAMP8 and the cleaved form of VAMP8. **c** WT*P.g*. and gingipains cleave endogenous VAMP8 in neonatal rat cardiomyocytes. The arrows indicate VAMP8 and cleaved forms of VAMP8

**Fig. 4:**
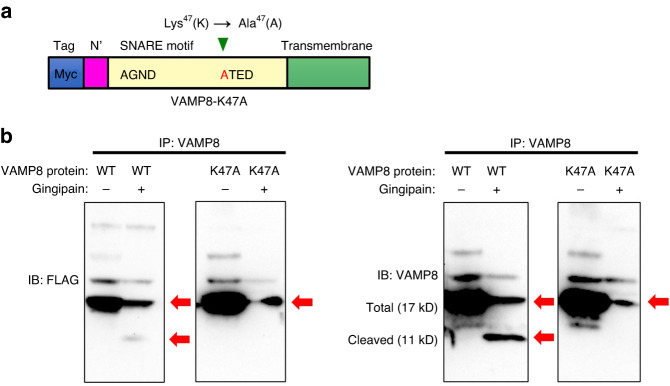
Gingipains cleave VAMP8 at its 47th lysine residue site. **a** Schematic diagram of the VAMP8-K47A protein. **b** Recombinant Flag-tagged VAMP8 protein and recombinant Flag-tagged VAMP8-K47A protein were immunoprecipitated with VAMP8 and incubated with gingipains followed by probing with the antibodies specific for FLAG or VAMP8. The gingipains cleave VAMP8 recombinant protein but do not cleave the VAMP8-K47A recombinant protein. The arrows indicate VAMP8 and the cleaved form of VAMP8

The fusion of lysosomal and autophagosomal membranes is facilitated by autophagic SNAREs. As VAMP8,^25^ a lysosomal SNARE protein, is a substrate for cysteine proteases.^26^ Moreover, a previous report revealed that caspase can cleave VAMP8 into its **AGND** and **KTED** sequences.^26^ Gingipain, a cysteine protease secreted by *P.g*., can cleave its substrates at arginine (Arg, R) or lysine (Lys, K) residues,^27^ suggesting that gingipains can cleave Lys47 residue in the KTED sequence of VAMP8 and inhibit lysosome–autophagosome fusion. To test this hypothesis, pull-down assays were conducted, which revealed that the recombinant VAMP8 protein physically interacted with gingipains (Fig. 3a). Cleavage assays also showed that the recombinant VAMP8 protein was cleaved by gingipains (Fig. 3b). In addition, to evaluate the effect of gingipains on endogenous VAMP8 in NRCMs, the presence of cleaved forms of VAMP8 was evaluated via immunoblotting in both WT*P.g*.-inoculated and gingipain-treated NRCMs. The cleaved form of VAMP8 was detected in both types of NRCMs. However, the cleavage form of VAMP8 was not observed in the NRCMs inoculated with Δ*P.g*. (Fig. 3c). Finally, it was observed that recombinant VAMP8-K47A mutant protein (Fig. 4a) was not cleaved by gingipains (Fig. 4b). Collectively, these results suggest that the gingipains released by *P.g*. cleave VAMP8 at its Lys47 residue.

### Gingipains impair the stability of the SNARE protein complex, STX17-SNAP29-VAMP8

**Fig. 5:**
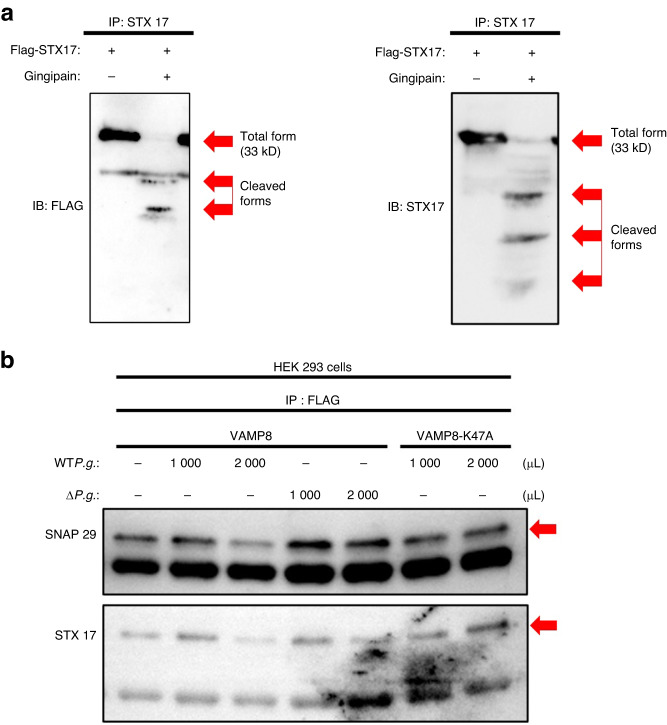
Gingipains impair the SNARE proteins complex. **a** Recombinant Flag-tagged STX17 protein was immunoprecipitated with an antibody specific to STX17 and incubated with gingipains followed by probing with antibodies specific for FLAG or STX17. The gingipains cleave the STX17 recombinant protein. The arrows indicate STX17 and cleaved forms of STX17. **b** Proteins from HEK293 cells were immunoprecipitated using an antibody specific to VAMP8 and detected with the use of an antibody specific to either SNAP29 or STX17

**Fig. 6:**
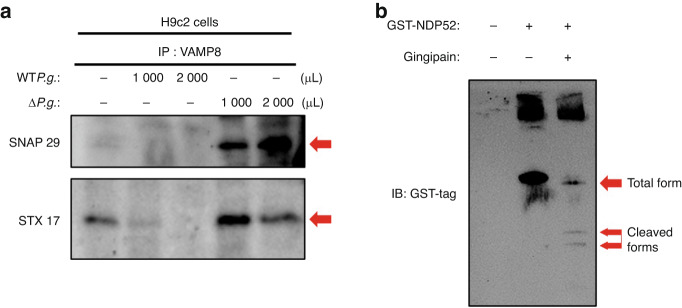
Gingipains impair the SNARE proteins complex and cleave NDP52. **a** Proteins from H9c2 cells were immunoprecipitated using an antibody specific to VAMP8 and were identified with an antibody specific to either SNAP29 or STX17. **b** Recombinant GST-tagged NDP52 was immunoprecipitated with a GST-tag-specific antibody and incubated with gingipains. The gingipains cleaved the NDP52 recombinant protein. The arrows indicate NDP52 and cleaved forms of NDP52

The fusion of the outer autophagosomal membrane with the late endosomal/lysosomal membrane is mediated by the interaction among syntaxin 17 (STX17), synaptosomal-associated protein 29 (SNAP29), and VAMP8.^28^ To gain an insight into the mechanism of gingipains in inhibiting the fusion of autophagosomes and lysosomes, we examined whether the gingipains disrupt the SNARE protein complex comprising STX17-SNAP29-VAMP8. Cleavage assays showed that gingipains could cleave the recombinant STX17 protein (Fig. 5a). Co-immunoprecipitation assays showed that the interactions between VAMP8 and SNAP29 and between VAMP8 and STX17 were significantly reduced in HEK293 cells transfected with VAMP8-WT in the presence of WT*P.g*. Conversely, there was no reduction in the interactions between VAMP8 and SNAP29 and between VAMP8 and STX17 in HEK 293 cells transfected with VAMP8-WT and inoculated with Δ*P.g*. or in HEK 293 cells transfected with VAMP8-K47A and inoculated with WT*P.g*. (Fig. 5b). We then examined the effect of gingipains on the interactions among STX17, SNAP29, and VAMP8 in H9c2 cells. To this end, we induced autophagy in H9c2 cells by starving them and inoculating them with either WT*P.g*. or Δ*P.g*. The interactions between VAMP8 and SNAP29 and between VAMP8 and STX17 decreased significantly in H9c2 cells inoculated with WT*P.g*., but did not decrease in those treated with Δ*P.g*. (Fig. 6a). Overall, these results suggest that gingipains from *P.g*. can cleave VAMP8 and STX17, thereby disrupting the interactions of STX17-SNAP29-VAMP8.

### Gingipains cleave NDP52 protein

We then explored the effect of *P.g*. on xenophagy, a type of autophagy that is critical to eliminate intracellular bacteria.^29^ Using gingipains, we performed a cleavage assay on NDP52, also known as calcium binding and coiled-coil domain 2 (CALCOCO2), which is a multifunctional autophagy adaptor crucial for xenophagy. Our results showed that administration of gingipains led to a significant reduction in NDP52 protein levels, along with the appearance of some fragments (Fig. 6b). This finding suggests that gingipains from *P.g*. is actively involved in NDP52 cleavage.

### Infection with *P.g*. decreases survival rate following MI in mice

**Fig. 7:**
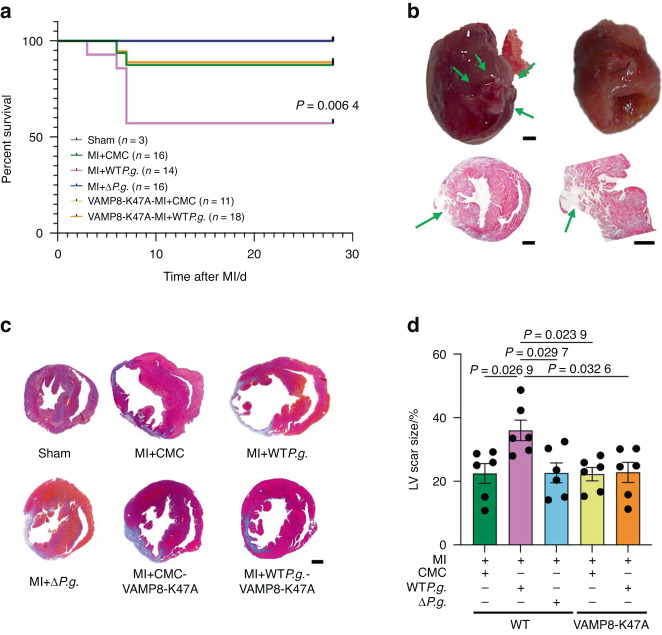
Infection with *Porphyromonas gingivalis* (*P.g*.) decreases the survival rate after MI. **a** The survival rate after MI of sham mice, MI + CMC mice, MI + WT*P.g*. mice, MI + Δ*P.g*. mice, VAMP8-K47A-KI + MI + CMC mice, and VAMP8-K47A + MI + *P.g*. mice. **b** Upper left, Arrows indicate rupture slits in the left ventricular free wall of a WT*P.g*.-inoculated MI mouse. *Upper right*, The heart of MI mouse (not ruptured) (scale bar = 1 mm). *Lower*, Representative images of myocardial slices of the ruptured heart (scale bar = 1 mm). Arrows indicate rupture slits. **c** Representative images of myocardial slices stained with Masson’s trichrome in the indicated groups of mice 28 days after MI (scale bar = 1 mm). **d** Quantitative analysis of the LV scar size in the indicated groups of mice at 28 days after MI (*n* = 6 for each group). The Kaplan–Meier survival analysis was used, and the survival rates were assessed using the log-rank test (**a**). Data are expressed as mean ± SEM. *P* values were obtained using one-way analysis of variance test followed by Turkey’s post-hoc test for multiple comparisons (**d**)

**Fig. 8:**
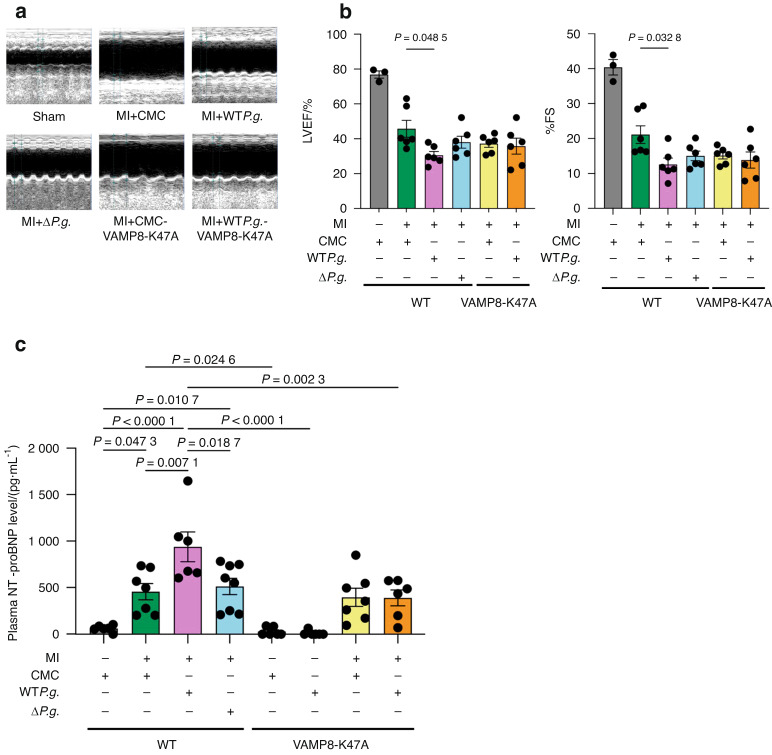
Echocardiographic analyses and plasma NT-proBNP level evaluations were conducted at 28 days after MI. **a** Representative images of echocardiograms at 28 days after MI. **b** Comparison of %EF and %FS in the indicated groups of mice (*n* = 3 for sham and *n* = 6 for MI + CMC, MI + WT*P.g*., MI + Δ*P.g*., VAMP8-K47A + MI + CMC, and VAMP8-K47A + MI + WT*P.g*. each). **c** Plasma levels of NT-proBNP 7 days after MI (*n* = 6 for sham, VAMP8-K47A-KI + WT*P.g*.-sham, VAMP8-K47A-KI + MI + CMC, and VAMP8-K47A-KI + MI + WT*P.g*., *n* = 7 for MI + CMC, MI + WT*P.g*., and VAMP8-K47A-KI + CMC-sham each and *n* = 8 for MI + Δ*P.g*.). Data are expressed as mean ± SEM. *P* values were obtained using one-way analysis of variance test followed by Turkey’s post-hoc test for multiple comparisons (**b**, **c**)

We used in vivo models to investigate the effect of *P.g*. infection on autophagy in post-MI heart failure. First, we generated VAMP8-K47A KI mice (VAMP8-K47A mice) using the clustered regularly interspaced short palindromic repeats (CRISPR)/Cas9 system (Fig. S1, S2). We then compared the effects of gingipains on the heart post-MI in wild-type (WT) and VAMP8-K47A mice. MI was induced in mice by performing permanent ligation of the left anterior descending (LAD) coronary artery in the following groups: WT*P.g*.-inoculated mice (MI + WT*P.g*.), Δ*P.g*.-inoculated mice (MI + Δ*P.g*.), carboxymethylcellulose (CMC)-inoculated mice (MI + CMC), CMC-inoculated VAMP8-K47A mice (VAMP8-K47A + MI + CMC), and WT*P.g*.-inoculated VAMP8-K47A mice (VAMP8-K47A + MI + WT*P.g*.) (Fig. S3a). At 28 days post-MI, ELISA showed a significant increase in anti-*P.g*. antibody titers in the MI + WT*P.g*., MI + Δ*P.g*., and VAMP8-K47A + MI + WT*P.g*. groups compared with the sham, MI + CMC, and VAMP8-K47A + MI + CMC groups (Fig. S3b). Kaplan–Meier analysis showed that the survival rate during the first 28 days after MI surgery was significantly lower in the MI + WT*P.g*. group (57.1%) than the MI + CMC (87.5%) and VAMP8-K47A + MI + WT*P.g*. (88.9%) groups (Fig. 7a). Postmortem analyses revealed that the cause of death in MI mice was LV rupture (Fig. 7b). The post-MI LV scar in the MI + WT*P.g*. group was significantly larger than that in the other groups(Figs. 7c, d). Echocardiographic analyses showed that the ejection fraction and FS of the LV were significantly lower in the MI + WT*P.g*. group than in the MI + CMC group (Figs. 8a, b, S4). NT-proBNP, an important biomarker that indicates the severity and prognosis of cardiac function, was significantly higher in the MI + WT*P.g*. group than in the other groups (Fig. 8c). These findings indicate that *P.g*. infection exacerbates the pathogenesis of MI in the ischemic myocardium and VAMP8 plays a critical role in this process.

### VAMP8 is cleaved and autophagy is suppressed in the hearts of WT*P.g*.-inoculated MI mice

**Fig. 9:**
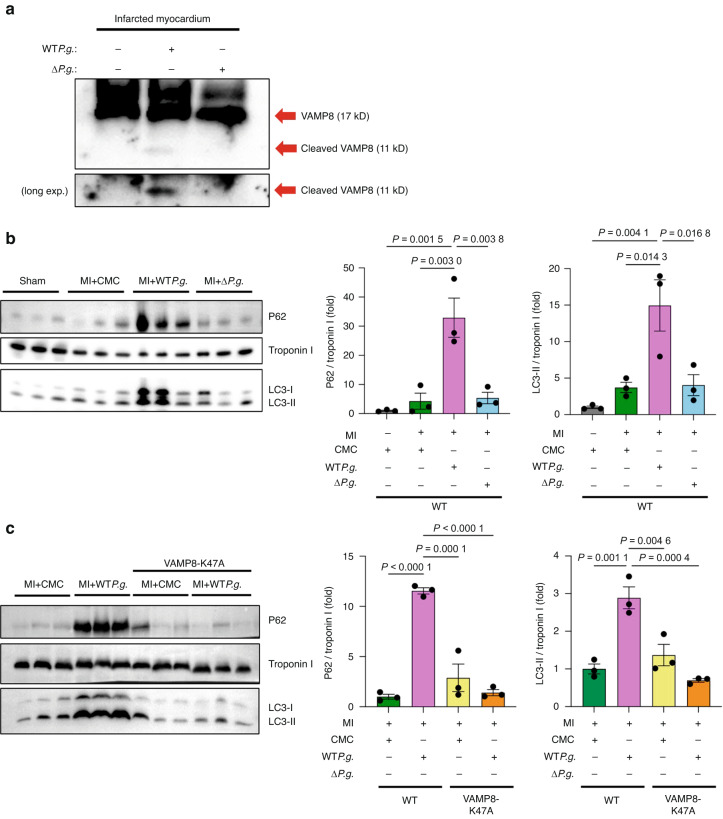
Infection with *P.g*. leads to vesicle-associated membrane protein 8 (VAMP8) cleavage and suppresses autophagy in MI mice hearts. (A) **a** Proteins were extracted from MI + CMC, MI + WT*P.g*., and MI + Δ*P.g*. mice hearts. Representative immunoblots with antibody specific to VAMP8 is shown. The arrows indicate VAMP8 and cleaved form of VAMP8. **b**. Proteins were extracted from the hearts of mice in the sham, MI + CMC, MI + WT*P.g*., and MI + Δ*P.g*. groups. Left, Representative immunoblots with antibodies specific to P62, Troponin I, and LC3. *Right*, Densitometric analysis of immunoblots are shown (*n* = 3 for each). **c**. Proteins were extracted from the hearts of mice in MI + CMC, MI + WT*P.g*., VAMP8-K47A-KI + CMC + MI, and VAMP8-K47A-KI + MI + WT*P.g*. groups. Left, Representative immunoblots with antibodies specific to P62, Troponin I, and LC3 are shown. *Right*, Densitometric analysis of immunoblots are shown (*n* = 3 for each). Results are representative of at least two independent experiments. Data are expressed as mean ± SEM. *P* values were determined by one-way analysis of variance test followed by Turkey’s post-hoc test for multiple comparisons (**b**, **c**)

To further understand the effect of gingipains on VAMP8 in post-MI hearts, we performed a protein extraction experiment on the hearts of mice inoculated with WT*P.g*. or Δ*P.g*. The results of the immunoblot analysis showed that the cleaved form of VAMP8 was present in the hearts of the MI + WT*P.g*. group, but not in those of MI + Δ*P.g*. mice (Fig. 9a). These results suggest that gingipains from *P.g*. can cleave VAMP8 in the infarcted myocardium. Next, we investigated whether autophagy is suppressed in the hearts of *P.g*.-inoculated MI mice. To examine the effect of *P.g*. infection on cardiac autophagy, we evaluated the levels of SQSTM1/P62 and LC3-II, which indicate autophagic degradation. Immunoblot analysis showed that the levels of SQSTM1/P62 and LC3-II were significantly higher in the hearts of the MI + WT*P.g*. group than those of the other groups, indicating an increase in the number of autophagosomes along with the disruption of the autophagic flux (Fig. 9b). On the other hand, the levels of SQSTM1/P62 and LC3-II were significantly lower in the hearts of the VAMP8-K47A + MI + WT*P.g*. group than those of the MI + WT*P.g*. group (Fig. 9c), indicating that VAMP8 cleavage and autophagy are suppressed in the hearts of the MI + WT*P.g*. group.

### *P.g*. infection suppresses the autophagy activity in myocardium

**Fig. 10:**
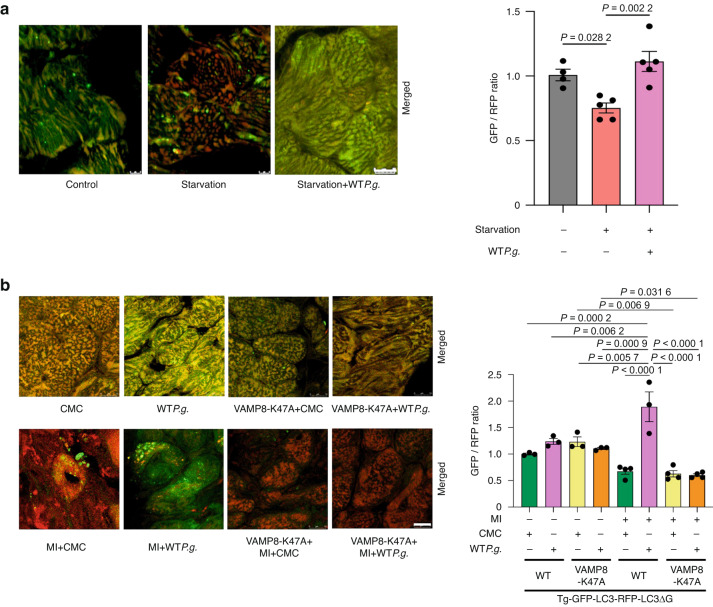
Infection with *P.g*. suppresses autophagy activity in myocardium. **a** Left, Representative fluorescence ratio images of GFP-LC3-RFP-LC3ΔG transgenic mice, 48 h post starvation-GFP-LC3-RFP-LC3ΔG transgenic mice, and 48 h post starvation and WT*P.g*.-inoculated-GFP-LC3-RFP-LC3ΔG transgenic mice hearts. *Right*, Quantitative analysis of the GFP/RFP fluorescence ratio are shown (*n* = 4 for control, *n* = 5 for starvation and starvation+*P.g*. groups). **b**. Left, Representative fluorescence ratio images of CMC-inoculated mice, WT*P.g*.-inoculated mice, CMC-inoculated VAMP8-K47A mice, WT*P.g*.-inoculated VAMP8-K47A mice, MI + CMC mice, MI + WT*P.g*. mice, VAMP8-K47A + MI + CMC mice, and VAMP8-K47A + MI + WT*P.g*. mice hearts 7 days after MI. *Right*, Quantitative analysis of the GFP/RFP fluorescence ratio are shown. Results are representative of at least two independent experiments. Data are expressed as mean ± SEM. *P* values were determined using one-way analysis of variance test followed by Turkey’s post-hoc test for multiple comparisons (**a**, **b**)

We then evaluated the effect of *P.g*. infection on autophagy in myocardium. To this end using transgenic mice expressing Tg-GFP-LC3-RFP-LC3ΔG, a fluorescent probe for evaluating the autophagic flux^30^ (Tg-tfLC3ΔG mice) (Fig. S5a, S5b). The Tg-tfLC3ΔG mice were divided into three groups: control, starvation, and starvation+WT*P.g*.-inoculation groups. Confocal microscopic analyses demonstrated that the GFP/RFP ratio of the starvation group was significantly lower than that of the control group, indicating the activation of autophagy. However, the GFP/RFP ratio of the starvation+WT*P.g*.-inoculation group was significantly higher than that of the starvation group, suggesting that inoculation of *P.g*. inhibits autophagy in vivo (Fig. 10a, S6a).

Next, VAMP8-K47A and Tg-tfLC3ΔG mice were crossed to generate VAMP8-K47A KI;Tg-GFP-LC3-RFP-LC3ΔG mice (VAMP8-K47A;Tg-tfLC3ΔG mice). We inoculated either CMC or WT*P.g*. and induced MI in both Tg-tfLC3ΔG and VAMP8-K47A; tfΔTg-LC3 mice to assess the degree of autophagic flux in the hearts 7 days after MI. The GFP/RFP ratio in MI + CMC mice was lower than that in the CMC-sham mice. The GFP/RFP ratio in VAMP8-K47A;Tg-tfLC3ΔG +MI + CMC mice was significantly lower than that in the VAMP8-K47A;Tg-tfLC3ΔG +CMC-sham mice. The GFP/RFP ratio in VAMP8-K47A;Tg-tfLC3ΔG +MI + WT*P.g*. mice is significantly lower than that in the VAMP8-K47A;Tg-tfLC3ΔG +WT*P.g*.-sham mice. These results indicate that MI activates autophagy. Conversely, the GFP/RFP ratio in MI + WT*P.g*. group was significantly higher than that in MI + CMC group, suggesting that WT*P.g*. inoculation suppressed autophagy. Furthermore, the GFP/RFP ratio in VAMP8-K47A;Tg-tfLC3ΔG +MI + WT*P.g*. group was significantly lower than that in MI + WT*P.g*. mice, indicating that VAMP8-K47A negated the suppression of autophagy caused by WT*P.g*. (Fig. 10b, S6b).

### *P.g*. infection causes hypertrophy in myocardium

**Fig. 11:**
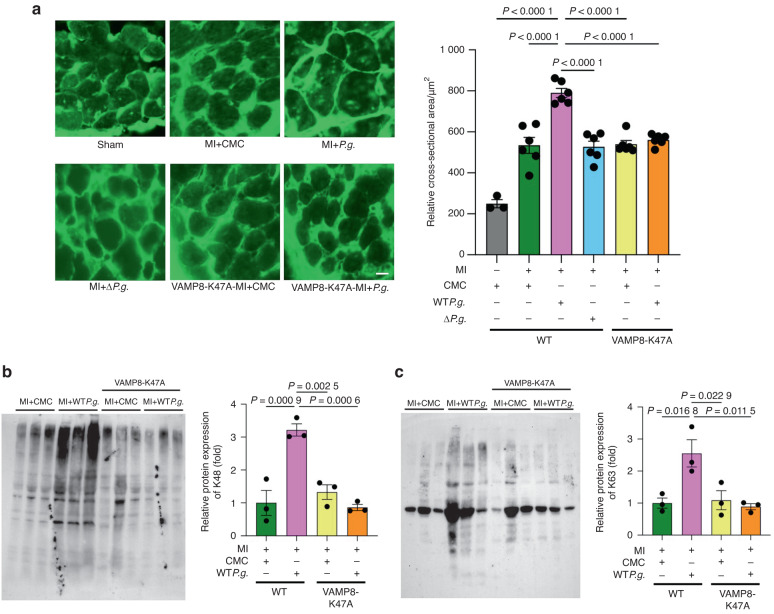
Infection with *P.g*. causes hypertrophy in myocardium. **a** Left, Representative images of myocardial slices stained with WGA in the indicated groups of mice 28 days after MI (scale bar = 10 μm). *Right*, Quantitative analysis of the cross-sectional area of the indicated groups are shown (*n* = 3 for control, *n* = 6 for other experimental groups). **b** Left, Representative immunoblots with ubiquitin-specific antibody specific to K48. *Right*, Densitometric analysis of immunoblots are shown (*n* = 3 for each group). **c**. Left, Representative immunoblots with ubiquitin-specific antibody specific to K63. *Right*, Densitometric analysis of immunoblots are shown (*n* = 3 for each group). Results are representative of at least two independent experiments. Data are expressed as mean ± SEM. *P* values were determined using one-way analysis of variance test followed by Turkey’s post-hoc test for multiple comparisons (**a**–**c**). *P* < 0.05 compared with MI + WT*P.g*. (**a**–**c**)

We also performed WGA staining to evaluate the size of myocardium 28 days after MI. The cross-sectional area of myocardium in the MI + WT*P.g*. group was significantly larger than that in the other groups, including the MI + Δ*P.g*. group, suggesting that gingipains from *P.g*. induced cardiomyocyte hypertrophy after MI. Conversely, the cross-sectional area of myocardium in the VAMP8-K47A + MI + WT*P.g*. group was significantly smaller than that in the MI + WT*P.g*. group, suggesting that VAMP8-K47A negates cardiomyocyte hypertrophy after MI caused by WT*P.g*. (Fig. 11a).

The role of the autophagic system is to not only degrade unnecessary components but also remove polyubiquitinated aggregated proteins.^31^ Therefore, we aimed to investigate the accumulation of polyubiquitinated proteins in the presence of impaired autophagy caused by *P.g*. infection in cardiomyocytes. To this end, we extracted proteins from the hearts of MI + CMC, MI + WT*P.g*., VAMP8-K47A + MI + CMC, and VAMP8-K47A + MI + WT*P.g*. groups and performed immunoblot analyses. The results showed that the hearts from the MI + WT*P.g*. group had higher levels of polyubiquitinated proteins than those in the other groups, whereas those of VAMP8-K47A + MI groups showed no such increase (Fig. 11b, c). These results indicate that WT*P.g*. infection in mice disrupted autophagy, leading to cardiomyocyte hypertrophy and accumulation of polyubiquitinated proteins in the infarcted myocardium.

## Discussion

The key findings of this study can be summarized as follows: (1) *P.g*. invades cardiomyocytes; (2) gingipains produced by *P.g*. cause serious damage to cardiomyocytes; (3) gingipains inhibit autophagosome–lysosome fusion, thereby suppressing the autophagic flux; (4) gingipains physically interact with and cleave VAMP8 at Lys47; (5) gingipains also cleave STX17, impairing the stability of the STX17–SNAP29–VAMP8 complex; (6) gingipains can cleave NDP52; (7) WT*P.g*. infection in MI mice worsens their survival after MI by increasing the incidence of cardiac rupture; however, the incidence of cardiac rupture was reduced in VAMP8-K47A mice, thereby improving survival; (8) *P.g*. infection in vivo leads to the cleavage of VAMP8 and suppression of autophagy; and (9) *P.g*. infection induces cardiomyocyte hypertrophy and accumulation of polyubiquitinated proteins.

Normally, autophagy properly occurs in the heart after MI.^32^ However, our results indicated that although autophagy was activated after MI in mice inoculated with CMC, it was suppressed after MI in mice inoculated with WT*P.g*. The observed imbalance between autophagosome formation and lysosomal degradation may result in excessive autophagosome accumulation, which can subsequently lead to cellular dysfunction and even death.^33^ Based on these findings, it can be inferred that cellular dysfunction and death caused by the accumulation of autophagosomes may contribute to cardiac rupture. Consistently, previous studies have shown that the suppression of the autophagy machinery leads to cardiac rupture after MI.^34,35^ Furthermore, our current study demonstrated that even in the presence of *P.g*. infection, low incidences of cardiac rupture and cardiomyocyte hypertrophy were observed, and autophagy was activated in VAMP8-K47A mice. This suggests that Lys47 plays an important role in mitigating the adverse effects of *P.g*. infection in MI mice. The autophagosome–lysosome fusion is initiated by the recruitment of STX17 to the outer membrane of completed autophagosomes and subsequent fusion with the SNARE protein, VAMP8, located on the lysosomal membrane, resulting in the formation of an autolysosome.^28^ Previous research has shown that *P.g*. can prevent degradation via autophagy, as they can survive in autophagosome-derived vacuoles and prevent autophagosome–lysosome fusion.^11^ The virulence factors of *P.g*., gingipains, exert similar effects as the *L. pneumophila* effector Lgp1137, a serine protease, by binding to and cleaving STX17, thereby inhibiting autophagy.^36^ Therefore, in theory, the cleavage of VAMP8 in the nontransmembrane region should significantly impair autophagosome–lysosome fusion. Consistently, our recent findings showed that gingipains can cleave VAMP8 at Lys47, weaken the STX17–SNAP29–VAMP8 complex, inhibit autophagosome–lysosome fusion, and lead to the accumulation of polyubiquitinated proteins. Furthermore, we observed the gingipain-mediated cleavage of STX17. Evidence suggests that selective autophagy can degrade invading pathogens, such as bacteria, via xenophagy. To initiate xenophagy, invading pathogens are first tagged with ubiquitin, which allows them to be recognized and engulfed by ubiquitin-binding protein adaptors, such as sequestosome 1 (SQSTM1/P62), a neighbor of BRCA1 gene 1 (*NBR1*), and NDP52. Next, the pathogens are delivered for degradation within the assembly of autophagosomes.^37^ NDP52, a binding adaptor, plays a critical role in the selective autophagic degradation of invading pathogens. However, some pathogens have developed various mechanisms to evade autophagic degradation. They evade autophagy by inhibiting the signaling pathways that lead to autophagy induction, masking themselves with host proteins to avoid recognition by the autophagy machinery, interfering with the autophagy process to evade targeting, or blocking the autophagosome–lysosome fusion.^17^ Dorn et al. have shown that *P.g*. can enter host cells and evade cellular defense mechanisms, mainly by avoiding its degradation in lysosomes.^11^ In Coxsackievirus B3 infection, the viral protein 3 C cleaves NDP52, resulting in the formation of a stable C-terminal fragment that retains the full-length gene function of NDP52.^38^ In this study, we confirmed that *P.g*. cleaves NDP52 into smaller fragments, but further investigation is warranted to determine the effect of this cleavage on NDP52 function and potential suppression of xenophagy.

Our current study demonstrated that gingipains play a role in cleaving and inactivating VAMP8, STX17, and NDP52 proteins, suppressing both autophagy and xenophagy and enabling *P.g*. to reside within host cells. Based on these findings, we hypothesized that the destruction of specific proteins by gingipains is a strategy employed by *P.g*. to enhance its pathogenicity in periodontal tissue. Indeed, previous studies have revealed that gingipains directly contribute to the degradation of periodontal tissue by breaking down the extracellular matrix components, such as collagen and fibronectin.^39,40^ Furthermore, gingipains are known to impair immune defense mechanisms by degrading immunoglobulins and the complement system.^41,42^ However, as all protein substrates targeted by gingipains have not yet been elucidated, further investigation is warranted to test our hypothesis. Further, as the current study primarily aimed to elucidate the mechanism by which gingipains hinder xenophagy through the inhibition of autophagosome–lysosome fusion, a separate future study is warranted to thoroughly investigate the association between gingipains and NDP52, a regulatory factor that facilitates xenophagy by labeling bacteria and promoting engulfment by autophagosomes.

When studying the effects of a specific pathogen such as *P.g*., it is important to minimize interference from other commensal bacteria. To do this, we administered trimethoprim and sulfamethoxazole to specific pathogen-free mice for 10 days prior to inoculation with *P.g*.^43^ Then, in order to mitigate the effects of antibiotics on *P.g*. after administration, we waited for the antibiotics to be naturally metabolized and cleared from the mice’s body. As the typical half-life of trimethoprim in mice is 4 to 6 h, it could take approximately 24 to 36 h for the drug to be cleared from the body after administration is stopped. Similarly, the half-life of sulfamethoxazole in mice is typically 5 to 8 h, it could take approximately 30 to 48 h for sulfamethoxazole to be cleared from the mice’s body after the last dose.

Generally, it is necessary to remove the antibiotics from the culture media prior to bacterial inoculation in order to assess the effect of bacterial infection in cells. On the other hand, because penicillin and streptomycin have relatively weak antibacterial activity against *P.g*., gentamicin and metronidazole are typically supplemented in the medium to effectively eradicate *P.g*.^44^ Furthermore, as our experiment was designed to evaluate the effect of invading *P.g*. on cardiomyocytes, we inoculated *P.g*. into cardiomyocytes with penicillin and streptomycin in the culture medium. Indeed, studies have shown that *P.g*. invading cultured cells can survive even in the presence of antibiotics.^45^ Consistently, we have previously shown that *P.g*. invade cultured cardiomyocytes despite the presence of penicillin and streptomycin in the culture media.^19^

It is important to consider a potential limitation that *P.g*. impairs post-infarcted myocardium by inhibiting the autophagic flux. To validate these findings, further in vivo experiments, such as rescue experiments aimed at reactivating the autophagic flux in WT*P.g*.-inoculated MI model mice, should be conducted.

Another limitation of this study is the lack of investigation of the effect of gingipains on the viability of *P.g*. within cardiomyocytes. In our study, we exclusively focused on assessing the impact of gingipains on cardiomyocyte viability following *P.g*. inoculation. However, to enhance the reliability of these findings, it is imperative to evaluate the potential influence of gingipain deficiency on the survival of *P.g*. within host cells in future studies.

In conclusion, invasion of the ischemic myocardium by *P.g*. leads to cleavage of VAMP8 at Lys47, thereby impairing autophagosome–lysosome fusion and suppressing autophagic activity. This results in the accumulation of polyubiquitinated proteins and a decline in cardiac function, ultimately increasing the incidence of cardiac rupture. Our study sheds light on the potential mechanisms through which *P.g*. infection may contribute to the viability of post-MI myocardium and elaborates the crucial role of autophagy in maintaining cardiac function. These findings provide new insights into the link between PD and MI and suggest targeting *P.g*. infection and enhancing autophagic function as a potential therapeutic approach for reducing the risk of MI and improving outcomes in patients with PD.

## Materials and methods

### Animals

Male C57BL/6 J WT mice (7-week-old; body weight, 20–25 g) were received from Japan Clea, Co. (Tokyo, Japan). Mice were housed under 12 h light/dark cycles at room temperature. All mice had free ad libitum access to normal diet and water. Animal care procedure and all experiments were performed in accordance with the Tokyo Medical and Dental University Guide for the Care and Use of Laboratory Animals (Permit Number: A2022–096C and G2019-038C5) and Guide for the Care and Use of Laboratory Animals published by the US National Institutes of Health.

Mice were deeply anesthetized with an intraperitoneal injection of a cocktail (medetomidine-midazolam-butorphanol (MMB); 0.1 mL per 10 g) comprising medetomidine (Domitor; Nippon Zenyaku Kogyo Co., Ltd., Fukushima, Japan; 0.75 mg·kg^-1^), midazolam (Dormicum; Maruishi Pharmaceutical, Osaka, Japan; 4 mg·kg^-1^), and butorphanol (Vetrophale; Meiji Seika Pharma CO., Ltd., Tokyo, Japan; 5 mg·kg^-1^). Euthanasia was performed by intraperitoneally administering an overdose of MMB.

### Genetically modified mice

The gRNA for the mouse *Vamp8* gene, donor oligo containing K47A (AAG to GCG) mutation sites, and Cas9 mRNA were introduced into mouse eggs to produce vesicle-associated membrane protein 8 (VAMP8)-K47A mutants. F0 founder animals were detected using polymerase chain reaction (PCR) followed by sequence analysis using the following primers: 5’-CAAGGGAGGAGTGACACCTGACCAC-3’ and 5’-ATCCAACCACTCACTGGGCTCTCTAC-3’.

To generate Tg-GFP-LC3-RFP-LC3ΔG mice (Tg-tfLC3ΔG mice), frozen sperm from C57BL/6J-Tg (CAG-GFP/LC3/RFP/LC3 < *>deltaG)2Nmz mice was provided by the RIKEN RBC through the National BioResource Project of the MEXT/AMED, Japan. We obtained the first generation of GFP-LC3-RFP-LC3ΔG heterozygous mice by external fertilization. The transgenic strains were identified by performing PCR using the following primers: 5’-CCTACAGCTCCTGGGCAACGTGC-3’; 5’-GTACCACCACACTGGGATCCTTAG-3’; 5’-CTAGGCCACAGAATTGAAAGATCT-3’; and 5’-GTAGGTGGAAATTCTAGCATCATCC-3’.

To generate VAMP8-K47A;Tg-tfLC3ΔG mice, VAMP8-K47A KI mice were bred in house with Tg-tfLC3ΔG mice.

### Bacterial growth

The WT*P.g*. strain ATCC33277 (WT*P.g*.) and gingipain-deficient *P.g*. strain KDP981 (Δ*P.g*.) were kindly provided by Dr. Keiko Sato. They were cultured on blood agar plates in the AnaeroPack system (Mitsubishi Gas Chemical. Co., Ltd. Tokyo, Japan) and were incubated at 37 °C for 2–3 days. Bacterial cells were then added to peptone yeast extract and incubated for 1 week. The bacterial concentration was estimated using a spectrophotometer at a wavelength of 660 nm and was standardized to 10^9^ CFU·mL^-1^.^46^

### Inoculation of periodontal pathogens

To evaluate the effects of *P.g*. on systemic organs other than periodontal tissue in mice, we employed the *P.g*. oral gavage method.^47^ This approach is widely used to obtain a less artificial infectious animal model, ensuring a more realistic representation of the biological response. Mice were administered with trimethoprim (0.4 mg·mL^-1^) and sulfamethoxazole (0.7 mg·mL^-1^) solutions (FUJIFILM Wako Pure Chemical. Co., Ltd. Tokyo, Japan), which were prepared by mixing the drugs with drinking water for 10 days, and only water was administered on the following 2 days. The mice were then inoculated once every 2 days with 0.1 mL of *P.g*. (1 × 10^9^ CFU·mL^-1^) dissolved in 2.5% carboxymethylcellulose (CMC; Nacalai Tesque, Inc., Kyoto, Japan) via oral gavage. Noninoculated mice were administered with 0.1 mL of 2.5% CMC alone.^48^ The level of anti-*P.g*.–specific IgG in the plasma before sacrificing the mice was determined using enzyme-linked immunosorbent assay (ELISA).^49^

### Extraction of gingipains from *P.g*

The A7A1–28 strain of *P.g*., which potentially produces gingipains,^50^ was used. Briefly, *P.g*. was inoculated into enriched tryptic soy broth and incubated at 37 °C under anaerobic conditions until the bacterial cells were sedimented. *P.g*. cells were centrifuged at 20 000 × *g* for 20 min at 4 °C. The supernatant was separated, and the precipitated protein was collected by centrifugation at 25 000 × *g* for 30 min at −10 °C. The suspension was then added to dialysis bags (MWCO, 12 000–14 000; Thermo Fisher Scientific, Waltham, MA, USA), which were placed in dithiopyridine disulfide buffer at 4 °C, followed by two changes gel filtration buffer at 4 °C. The dialyzed fraction was obtained by performing ultracentrifugation for 1 h at 100 000 × *g* at 4 °C and separated by ultrafiltration using an Amicon PM-30 membrane (Merk Millipore, Burlington, MA, USA).

### Experimental MI model

MI was induced as previously described.^51^ Mice were deeply anesthetized with an intraperitoneal injection of MMB (0.1 mL per 10 g). Overall, the mice were intubated using a small rodent ventilator (MiniVent 845, Harvard Apparatus, Holliston, MA). Left lateral thoracotomy was performed, and the LAD coronary artery was ligated using an 8/0 nylon suture after removing the pericardium. The chest was closed, antipamezole (0.75 mg·kg^−1^; Antisedan; Nippon Zenyaku Kogyo Co., Ltd.) was administered for reversal, and then the mice were removed from the respirator. The mice were allowed to recover on a warm surface. Sham mice underwent all procedures except for actual LAD occlusion. Some mice were examined until 28 days after MI to analyze survival. The other mice were examined until 7 days after MI and then sacrificed to obtain samples. All mice were necropsied to obtain evidence regarding post-MI cardiac rupture or heart failure as described previously.^52^

### Hemodynamic measurements and echocardiography

A tail-cuff system (BP-98A; Softron Co., Tokyo, Japan) was used to measure the arterial blood pressure and heart rate. Transthoracic echocardiography was carried out on mice that were administered MMB intraperitoneally (0.08 mL per 10 g body weight). The left ventricle function was assessed using an echocardiographic machine with a 14-MHz transducer (Toshiba, Tokyo, Japan). A two-dimensional targeted M- and B-modes echocardiogram was received, and left ventricular end-diastolic (LVDd) and end-systolic (LVDs) dimensions and left ventricular fractional shortening (LVFS) (LVFS = (LVDd − LVDs)/LVDd) were computed over three cardiac cycles according to the leading edge method described by the American Society for Echocardiography. The average of measurements from three consecutive cardiac cycles, performed off-line by two independent investigators, was calculated.

### Histopathologic examinations

The hearts were harvested immediately after sacrificing the mice on day 7 or 28 after MI induction. Midventricular slices of the heart were stained using hematoxylin and eosin stain, Mallory’s trichrome stain, and lectin obtained from *Triticum vulgars* (WGA; Sigma-Aldrich, St. Louis, MO, USA). The area of fibrosis represented by blue-stained collagen fibers was calculated using ImageJ. The average infarct size was defined as the average circumference of the infarcted portion and the normal area from the consecutive myocardial sections.^32^

### Measurement of plasma levels of NT-proBNP

At 7 days after MI, blood samples were collected and plasma NT-proBNP levels were measured using a mouse NT-proBNP ELISA kit (Novus Biologicals, Centennial, CO, USA) according to the manufacturer’s instructions.

### Preparation of primary neonatal rat cardiomyocytes (NRCMs)

Ventricular cardiomyocytes were isolated from 1- or 2-day-old neonatal Wistar rats using the Pierce Primary Cardiomyocyte Isolation Kit (Thermo Fisher Scientific), following a previously described method^53^ with minor modifications. To achieve NCRM purification, centrifugation was performed using a discontinuous Percoll (Sigma-Aldrich) gradient. The NRVM cultures contained >97–99% cardiomyocytes, as confirmed by immunofluorescence staining using the MF20 monoclonal antibody against sarcomeric myosin (Sigma-Aldrich). NCRMs were then incubated in Eagle’s minimum essential medium (Sigma-Aldrich) enriched with 5% calf serum (JRH Biosciences, Lenexa, KS, USA) for 24 h at 37 °C. NRCMs were treated with WT*P.g*., Δ*P.g*., or gingipains for 4 h at 37 °C.

### Cell culture

Human embryonic kidney (HEK) 293 T cells and H9c2 cells were obtained from the American Type Culture Collection (Manassas, VA, USA) and cultured in Dulbecco’s Modified Eagle Medium (DMEM; Sigma-Aldrich) enriched with 10% fetal bovine serum (Thermo Fisher Scientific), 100 U·mL^-1^ penicillin, and 100 mg·mL^-1^ streptomycin (Nacalai tesque, Kyoto, Japan) at 37 °C. For the starvation treatment, cells were rinsed with phosphate buffer saline (PBS) and were put in amino acid-free DMEM (FUJIFILM Wako Pure Chemical Co., Ltd., Osaka, Japan) without serum.

### Protein extraction from the myocardium and the cells

The infarct area of MI heart specimens was removed on day 7 after MI and homogenized in radioimmunoprecipitation assay (RIPA) buffer (containing 50 mmol·L^-1^ Tris-HCl, pH 6.8, 150 mmol·L^-1^ NaCl, 1 mmol·L^-1^ PMSF, 2 ng·mL^–1^ aprotinin, 1% Triton X-100, 1% SDS, and 1% sodium deoxycholate). HEK 293 T and H9c2 cells were washed with PBS and lysed with IGEPAL CA-630 buffer containing protease and phosphatase inhibitors (Sigma-Aldrich). The samples were centrifuged and supernatant was transferred to new tubes. Protein concentration was calculated using bicinchoninic acid (BCA) assay (Pierce Biotechnology, Rockford, IL, USA). Unused proteins were stored at −80 °C until further use.

### Transfection

Plasmid DNA of FLAG-VAMP8, FLAG-syntaxin 17 (Stx17), and FLAG-synaptosoma–associated protein 29 (SNAP29) was purchased from Addgene (Watertown, MA, USA). Plasmid DNA of FLAG-VAMP8-K47A was purchased from Vector Builder (Chicago, IL, USA). HEK 293 T cells (10 mL) were plated in a 10-cm dish 2 days before transfection. 1 mL Opti-MEM (Gibco, Billings, MT, USA), 10 μg plasmid DNA, and 20 μL FuGENE (Promega, Madison, WI, USA) were properly mixed and incubated for 15 min at room temperature. The medium of the HEK 293 T cells was aspirated. Next, the transfection complex and 5 mL DMEM were added dropwise to the cells and incubated at 37 °C. After 24 h, another 5 mL of DMEM was added and the cells were incubated for 24 h at 37 °C. The transfected cells were treated with either WT*P.g*. or Δ*P.g*. for 4 h at 37 °C.

### Immunoblotting

Equal amounts of protein from the infarcted area of hearts after 7 days of inducing MI, as well as NRCMs, HEK 293 cells, or H9c2 cells were separated using AnykD^TM^ precast sodium dodecyl sulfate–polyacrylamide gel electrophoresis (SDS–PAGE) gels (Bio-Rad Laboratories, Inc., Hercules, CA, USA). The separated proteins were transferred onto polyvinylidene difluoride (Immobilon-P; Merck Millipore, Burlington, MA, USA) or nitrocellulose (Bio-Rad) membranes, which were then incubated overnight with primary antibodies (anti-VAMP8, Abcam, ab76021, 1:1 000; anti-endobrevin, Santa Cruz Biotechnology [Dallas, TX, USA], sc-166820, 1:1 000; anti-p62, MBL [Nagoya, Japan], M162-3, 1:1 000; anti-LC3, MBL, M186-3, 1:1 000; anti-troponin I, Cell Signaling, #4002, 1:1 000; anti-SNAP29, Proteintech [Rosemont, IL, USA], 12704-1-AP, 1:500; and STX17, Proteintech, 17815-1-AP, 1:500). The membranes were then incubated with a secondary antibody (anti-rabbit or anti-mouse horseradish peroxidase [HRP]-conjugated antibodies, Cell Signaling) for 2 h, and the catalysis was promoted using SuperSignal West Dura Extended Duration Substrate (Thermo Fisher Scientific). Enhanced chemiluminescence using iBright Imaging systems was used to detect the proteins (Thermo Fisher Scientific).^25^

### Pull-down binding assays

Initially, VAMP8-modified 293 cells were washed with PBS and lysed with IGEPAL CA-630 buffer containing protease and phosphatase inhibitors. His-tagged Lys-gingipain recombinant protein (enQuireBioReagents, Littleton, CO, USA) was incubated with cobalt resin (Thermo Fisher Scientific) and IGEPAL CA-630 buffer at 4 °C for 2 h. The supernatant was removed and gingipain activation buffer^54^ (200 mmol·L^-1^ HEPES [pH 8.0], 5 mmol·L^-1^ CaCl_2_, and 20 mmol·L^-1^ L-cysteine, HCl solution) were added and incubated at 37 °C for 30 min. The samples were washed thrice with IGEPAL CA-630 buffer and 1× SDS sample buffer and processed for immunoblotting.

### Protein cleavage assay

VAMP8-, VAMP8-K47A-, or STX17-modified 293 cells were lysed with IGEPAL CA-630 buffer containing protease and phosphatase inhibitors. Protein concentration was determined using BCA assay, and the unused samples were stored at −80 °C until further use. Anti-FLAG M2 magnetic beads (Sigma-Aldrich) were rinsed thrice with IGEPAL CA-630 buffer and incubated with the samples for 2 h at 4 °C. The samples were washed thrice with high salt IGEPAL CA-630 buffer. The supernatants were then replaced with gingipain activation buffer. Gingipains were added and incubated for 30 min at 37 °C. After washing the sample thrice with IGEPAL CA-630 buffer, 1× SDS sample buffer was added to the beads. The obtained samples were then subjected to SDS–PAGE.

### Immunoprecipitation

NRCMs were lysed using RIPA buffer containing protease and phosphatase inhibitors. Next, VAMP8- and VAMP8-K47A-modified 293 cells and H9c2 cells were plated in a 10 mL volume in a 10-cm dish and incubated at 37 °C. After 2 days, the cells were rinsed with PBS, starved in amino acid-free DMEM, and then treated with either WT*P.g*. or Δ*P.g*. for 4 h at 37 °C. The cells were lysed with IGEPAL CA-630 buffer. Primary antibody (VAMP8) was covalently immobilized on protein A/G agarose (Thermo Scientific) for 1 h at 4 °C. Samples were incubated with immobilized antibody beads overnight at 4 °C. After immunoprecipitation, the samples were washed thrice with IGEPAL CA-630 buffer, followed by adding 1× SDS sample buffer. The obtained samples were then subjected to SDS–PAGE using specific primary antibodies and a conformation-specific secondary antibody that recognizes only native IgG (Cell Signaling, #5127, 1:2 000).

### NDP52 gingipains assay

GST–agarose beads were incubated with the GST–NDP complex (CALCOCO2 Recombinant Protein; Abnova [Taipei, Taiwan]) and then incubated with gingipains at 37 °C for 30 min. After washing the samples thrice with IGEPAL CA-630 buffer, the beads were collected. Proteins in the beads were then eluted using 1× SDS sample buffer, separated using SDS–PAGE and a nitrocellulose membrane, and incubated overnight with a primary antibody, GST-Tag monoclonal antibody (Proteintech, #66001-2-Ig, 1:500). The membrane was then incubated with a secondary antibody (anti-mouse or anti-rabbit HRP-conjugated antibodies; Cell Signaling) for 2 h, and the signal was developed with SuperSignal West Dura Extended Duration Substrate. Chemiluminescence was detected using iBright Imaging Systems.

### Mice starvation

In this study, 7-week-old Tg-tfLC3ΔG male mice were used. To promote systemic autophagy, mice were starved for 48 h.^55^ During the starvation period, the mice had free access to water.

### Cell viability assays

Cell viability was assessed using the Cell Counting Kit-8 (CCK-8, Dojindo Molecular Technology, Kumamoto, Japan). In brief, H9c2 cells and NRCMs (1 × 10^6^ cells per 100 μL) were seeded into 96-well dishes. After 24 h, the cells were incubated under hypoxic conditions at 37 °C for 24 h. Next, the cells were inoculated with either WT*P.g*. or Δ*P.g*. (1 × 10^8^ CFU·mL^–1^) and incubated for 1 h. The cell viability assays were performed according to the manufacturer’s instructions in the CCK-8. The experiments were conducted in triplicates.

### Detection of GFP-LC3 or mRFP-GFP-LC3

Adenovirus-harboring tandem fluorescent mRFP-GFP-LC3 was generated using a previously described method.^55^ NRCMs were grown on gelatinized coverslips, and transduction was performed using mRFP-GFP-LC3. After 48 h, the growth medium was replaced by phenol red-free medium. Then, either WT*P.g*. or Δ*P.g*. (1 × 10^8^ CFU·mL^–1^) was added and the samples were incubated for 1 or 4 h. For tissue samples, Tg-tfLC3ΔG mice were anesthetized with MMB (0.1 mL per 10 g body weight), and thoracotomy was performed. The hearts were excised and immediately embedded in Tissue-Tek OCT compound (Sakura Finetechnical Co., Ltd., Tokyo, Japan), which were stored at −80 °C. Next, 5–7 μm-thick samples were sectioned using a cryostat, air dried for 30 min, and stored at 4 °C. The fluorescence of GFP-LC3 or mRFP-GFP-LC3 was observed under a fluorescence microscope.^55,56^

### TEM analysis

To detect *P.g*. in NRCMs, the Click-iT^TM^ Cell Reaction Buffer Kit (Thermo Fisher Scientific) was used according to the manufacturer’s instructions. Briefly, *P.g*. was cultured with alkyne-conjugated N-acetylmuramic acid and was inoculated in NRCMs. To prepare electron microscopy specimens, *P.g*. was labeled with azide-conjugated gold nanoparticles. The samples were fixed in a 2.5% glutaraldehyde solution for 1 h, rinsed in 1% bovine serum albumin (BSA), and the Click-iT ^TM^ reaction cocktail was added. The samples were incubated at room temperature for 30 min away from light, followed by washing the samples with 1% BSA and fixing them in 0.3% osmium tetroxide (OsO_4_) dissolved in 0.1 mol·L^−1^ PBS for 17 min. They were then washed with 0.1 mol·L^−1^ PBS, dehydrated in ethanol, and stained with 2% uranyl acetate in 70% ethanol for 1 h at 4 °C. Subsequently, the samples were dehydrated in a graded series of ethanol and placed in Epon 812 (TAAB, Aldermaston, United Kingdom) for 48 h. Ultrathin (70–80 nm) sections were placed on copper grids and were observed under a transmission electron microscope (H-7011; Hitachi, Tokyo, Japan).

### Confocal fluorescence microscopic analysis

To detect *P.g*. in the cultured cardiomyocytes, *P.g*. was labeled with Acetoxymethyl Ester (BCECF-AM), as previously described.^57^ NRCMs were seeded into a 24-well circular cover-glass plate and grown overnight. Prior to inoculation, *P.g*. was incubated with BCECF-AM in PBS at 37 °C for 30 min. Then, NRCMs were inoculated with BCECF-labeled *P.g*. at 37 °C for 30 min in the dark and the cells were washed thrice with PBS. The cardiomyocytes were fixed with 4% formaldehyde in PBS for 10 min at room temperature. Cells were then permeabilized with 0.2% Triton X-100 for 10 min. TRITC-phalloidin (50 μg·mL^-1^) was placed on coverslips for 45 min. The nuclei were stained with 4′,6-diamidino-2-phenylindole (DAPI, Cosmo Bio Co., Ltd., Tokyo, Japan), and the coverslips were set on microscope slides. Confocal images were obtained using a confocal fluorescence microscope (TCS SP8; Leica, Wetzlar, Germany).

### Statistical analysis

Obtained data were expressed as mean ± SEM. Kaplan–Meier survival analysis was used, and the survival rates were compared using the log-rank test. Other statistical analyses were done using one-way analysis of variance test followed by Tukey’s post-hoc test for multiple comparisons. A *P*-value of <0.05 was considered to indicate statistical significance, and GraphPad Prism (GraphPad Software, San Diego, CA, USA) was used for the analyses.

### Supplementary information

Supplemental Figures

## Data Availability

All data associated with this study are presented in the manuscript or are available from the corresponding author upon reasonable request.
