# Supplementary material for: Porphyromonas gingivalis, a periodontal pathogen, impairs post-infarcted myocardium by inhibiting autophagosome–lysosome fusion
Source: Int J Oral Sci. 2023 Sep 18;15:42. doi: 10.1038/s41368-023-00251-2 (PMC10507114; doi:10.1038/s41368-023-00251-2)
Supplement: Supplementary file 1 — Supplemental Figures [file 41368_2023_251_MOESM1_ESM.pdf]

Supplemental Materials

Porphyromonas gingivalis, a periodontal pathogen, impairs post-infarcted myocardium by inhibiting autophagosome–lysosome fusion

Yuka Shiheido-Watanabe[1], Yasuhiro Maejima[1], Shun Nakagama[1], Qintao Fan[1], Natsuko Tamura[1] and Tetsuo Sasano[1]

[1] Department of Cardiovascular Medicine, Graduate School of Medical and Dental Sciences, Tokyo Medical and Dental University, Tokyo, Japan

Fig. S1

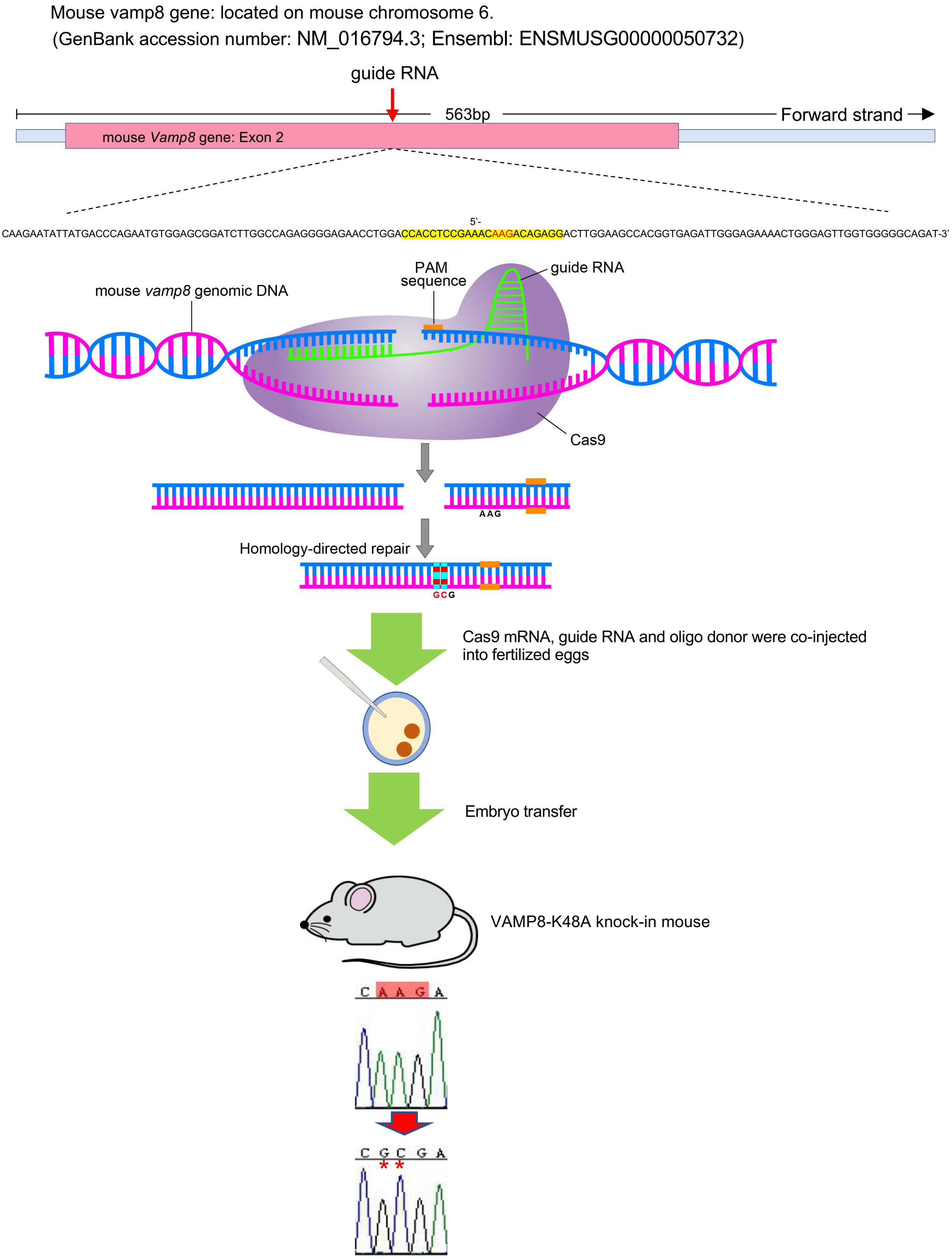

Fig. S1 Schematic diagram of vesicle-associated membrane protein 8 (VAMP8)-K47A mouse. C57BL/6J mouse model with a point mutation (K47A; AAG to GCG) at mouse *Vamp8* locus by CRISPR/Cas-mediated genome engineering.

Fig. S2

a

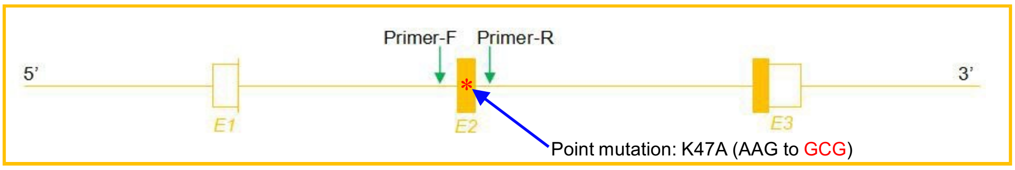

b

PCR Region1 (Annealing Temperature 60.0 ℃):

Mouse Vamp8(K47A)-F: CAAGGGAGGAGTGACACCTGACCAC

Mouse Vamp8(K47A)-R: ATCCAACCACTCACTGGGCTCTCTAC

Mutant allele: 563 bp; Wild type allele: 563 bp

PCR Mixture for PCR Region1

Component	x1	
Mouse tail genomic DNA	1.5	μl
Forward primer (10 μM)	1	μl
Reverse primer (10 μM)	1	μl
dNTPs (2.5 mM)	1.5	μl
10×PCR Buffer (Mg2+ Plus)	3	μl
TaKaRa Taq HS (5 U/μl)	0.2	μl
ddH_2O	21.8	μl
Total	30	μl

Cycling Condition :

Step	Temp.	Time	Cycles
Initial denaturation	94 °C	5 min	
Denaturation	94 °C	30 s	35 x
Annealing	60 °C	30 s	35 x
Extension	72 °C	60 s/kb	35 x
Additional extension	72 °C	5 min	

c

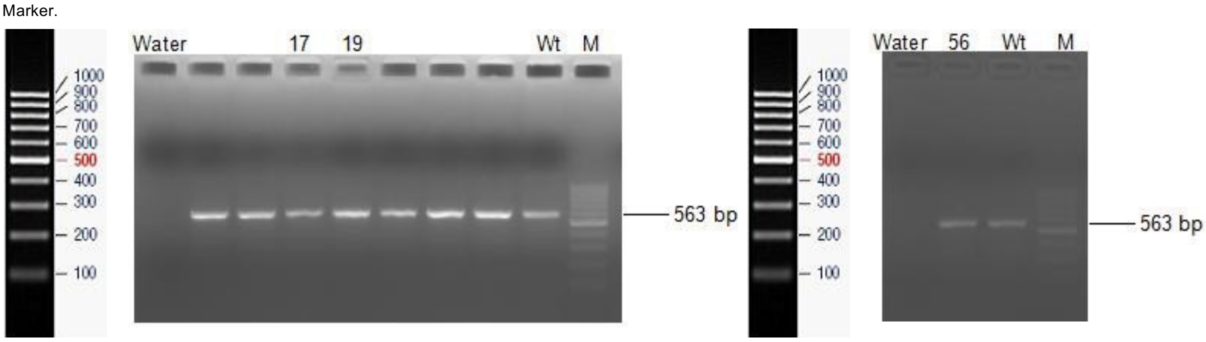

d

Sequencing Primer for PCR product: Sequence primer (Forward Sequencing): ACGTGCTTGCATCGAAGTGAA

Sequencing Results: Mouse ID:19 (two point mutations (AAG to GCG) happened in two alleles)

5'-AGAGGGGAGAACCTGGACCACCTCCGAAACGCGACAGAGGACTTGGAAGCCACGGTGAGA-3'(AAG>GCG)

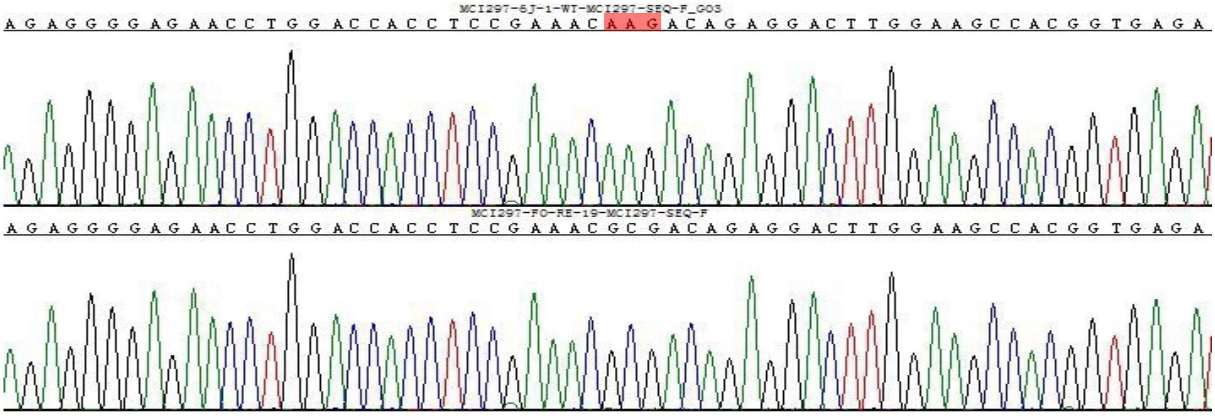

Fig. S2 Genotyping for VAMP8-K47A knock-in (KI) mouse. a. Schematic diagram of genotyping strategy for VAMP8-K47A-KI mouse. The K47A (AAG to GCG) mutation sites in donor oligo is induced into exon 2. **b.** PCR conditions for genotyping for VAMP8-K47A-KI mouse. **c.** Representative gel images of PCR products for genotyping. **d.** Representative DNA sequence images of wild type mouse (Upper) and VAMP8-K47A-KI mouse (Lower).

Fig. S3

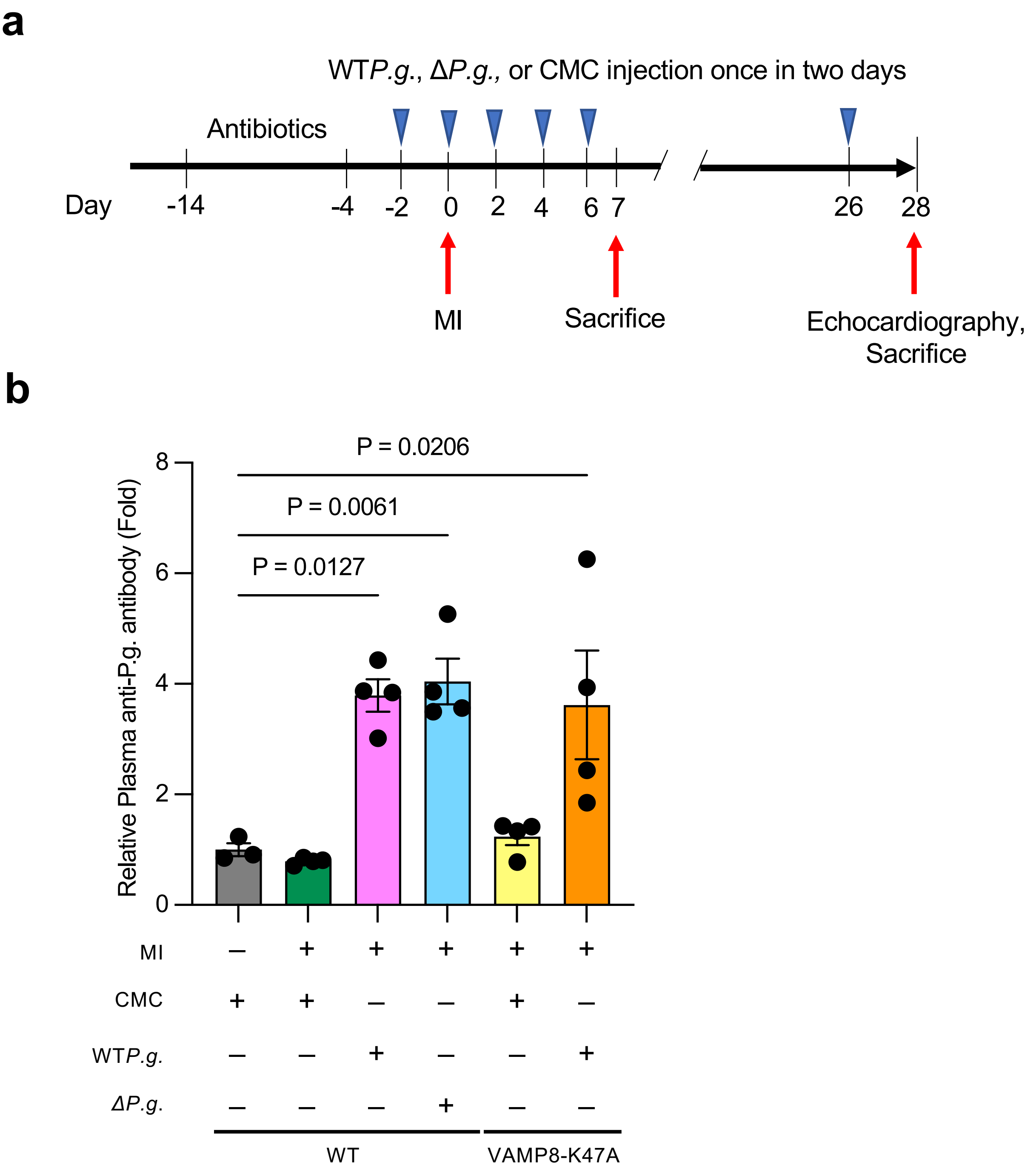

Fig. S3 Experimental design and supplemental data of MI models. a. Schematic diagram of the experimental design for injection of WT*P.g.* or Δ*P.g.* and MI. Briefly, mice were treated with antibiotic (ATB) in the drinking water *ad libitum* for 10 days, followed by 2 days of antibiotic-free period. Then, the mice were inoculated via oral gavage with either 1×10^9 CFU/mL of WT*P.g.* or Δ*P.g.* in 2.5% CMC or CMC only. **b.** Plasma levels of anti-*P.g.* specific IgG antibodies 28 days after MI (n=3 for sham and n=4 for MI+CMC, MI+WT*P.g.*, MI+Δ*P.g.*, vesicle-associated membrane protein 8 (VAMP8)-K47A+MI+CMC, and VAMP8-K47A+MI+*P.g.* each). Results are representative of at least two independent experiments.

Fig. S4

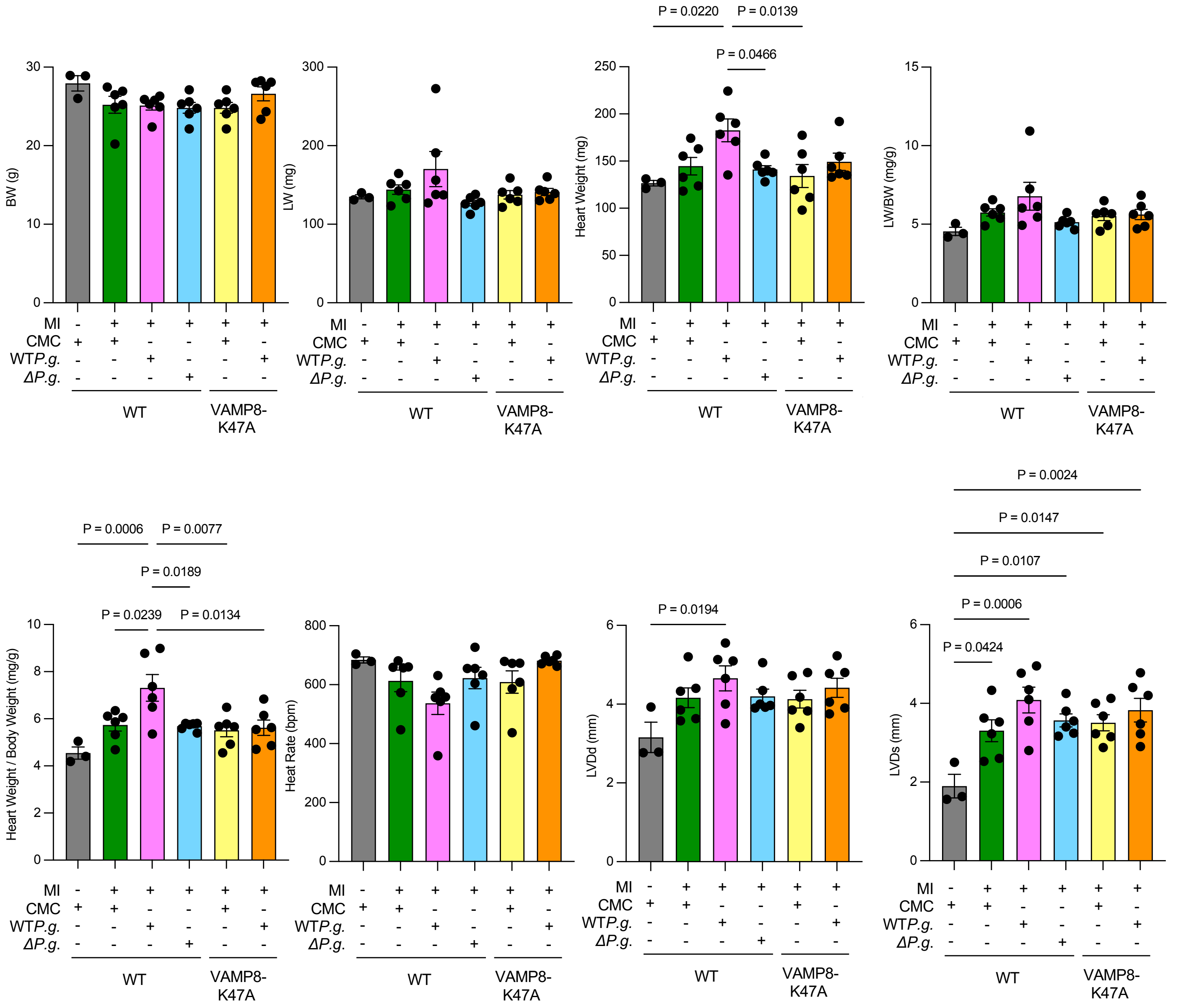

	Sham	MI+CMC	MI+WTP.g.	MI+ΔP.g.	VAMP8-K47A-MI+CMC	VAMP8-K47A-MI+WTP.g.
n	3	6	6	6	6	6
BW (g)	27.95 ± 0.97	25.21 ± 1.08	25.21 ± 0.58	24.83 ± 0.68	24.32 ± 1.82	26.61 ± 0.89
HW (mg)	126.5 ± 2.95	144.58 ± 9.33	182.6 ± 12.08	141.15 ± 3.98	134.22 ± 12.23	149.2 ± 9.15
LW (mg)	135.03 ± 2.6	144.22 ± 6.02	170.25 ± 22.44	127.03 ± 3.58	137.52 ± 5.30	141.12 ± 4.43
HW/BW (mg/g)	4.54 ± 0.26	5.73 ± 0.25	7.31 ± 0.57	5.69 ± 0.07	5.52 ± 0.28	5.62 ± 0.32
LW/BW (mg/g)	4.84 ± 0.18	5.75 ± 0.23	6.79 ± 0.89	5.13 ± 0.15	5.74 ± 0.26	5.35 ± 0.32
SBP (mmHg)	103.33 ± 4.67	94.67 ± 4.85	99 ± 9.81	111.5 ± 9.26	113.33 ± 4.88	102.5 ± 4.36
MBP (mmHg)	81 ± 1.53	70.5 ± 3.04	68.5 ± 8.3	79 ± 7.76	81.5 ± 7.8	79.33 ± 3.69
DBP (mmHg)	69.67 ± 0.33	58.67 ± 3.40	53 ± 7.81	63 ± 7.91	71.5 ±6.74	67.67 ± 3.54
HR (bpm)	684 ± 10.41	613.5 ± 36.68	537 ± 37.78	622.5 ± 36.32	609.17 ± 37.89	681.67 ± 6.04
FS (%)	40.38 ± 2.21	21.13 ± 2.52	12.6 ± 1.78	15.01 ± 1.38	15.11 ± 0.97	13.86 ± 2.27
EF (%)	76.87 ± 2.11	45.77 ± 4.88	30.57 ± 2.14	38.07 ± 3.42	37.22 ± 2.05	35.8 ± 4.55
LVDd (mm)	3.16 ± 0.38	4.16 ± 0.25	4.65 ± 0.32	4.2 ± 0.18	4.13 ± 0.22	4.42 ± 0.24
LVDs (mm)	1.9 ± 0.30	3.31 ± 0.28	4.08 ± 0.33	3.57 ± 0.16	3.51 ± 0.20	3.83 ± 0.30

Fig. S4 Echocardiographic data and postmortem pathologic measurements of MI models.

Fig. S5

a

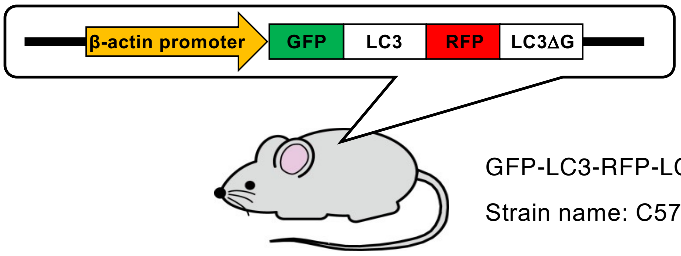

GFP-LC3-RFP-LC3ΔG transgenic autophagy reporter mouse

Strain name: C57BL/6J-Tg(CAG-GFP/LC3/RFP/LC3<*>deltaG) 2Nmz

b

	primer_name	primer_seq	length
1	5CAG	CCTACAGCTCCTGGGCAACGTGC	23
2	delta G	GTACCACCACACTGGGATCCTTAG	24
3	oIMR0042	CTAGGCCACAGAATTGAAAGATCT	24
4	oIMR0043	GTAGGTGGAAATTCTAGCATCATCC	25

Reaction Components

	Conc	Run 1
H_2O		0.8
PCR buffer	2 X	10.0
dNTP	2.0 mM	4.0
primer 1	10 pmol/μl	1.0
primer 2	10 pmol/μl	1.0
primer 3	10 pmol/μl	1.0
primer 4	10 pmol/μl	1.0
DNA		1.0
Taq	1 U/μl	0.2
	Total	20

Reaction Conditions

step	temp (ºC)	time (sec)	note
1	94	120	
2	98	10*	
3	60	30*	
4	68	240*	*Repeat for 30 cycles

c

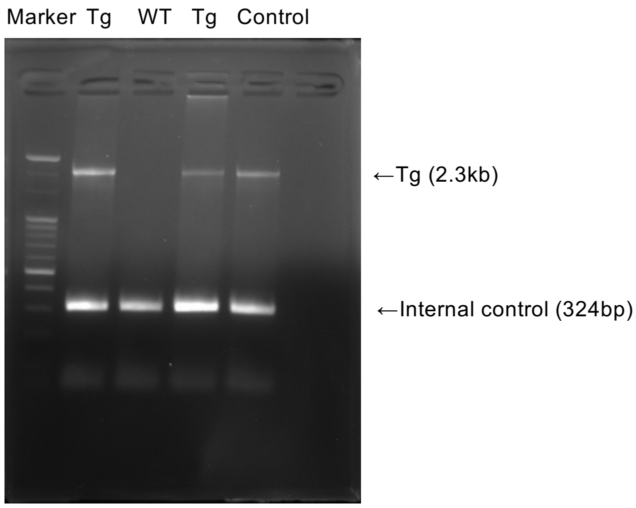

Fig. S5 Supplemental information regarding the experiments with Tg-GFP-LC3-RFP-LC3ΔG (Tg-tfLC3ΔG) mice. a. The scheme of C57BL/6 Transgenic-mouse harboring the sequence for GFP-LC3-RFP-LC3ΔG (Tg-tfLC3ΔG mouse). **b.** PCR conditions for genotyping for Tg-tfLC3ΔG mouse. **c.** Representative gel images of PCR products for genotyping.

Fig. S6

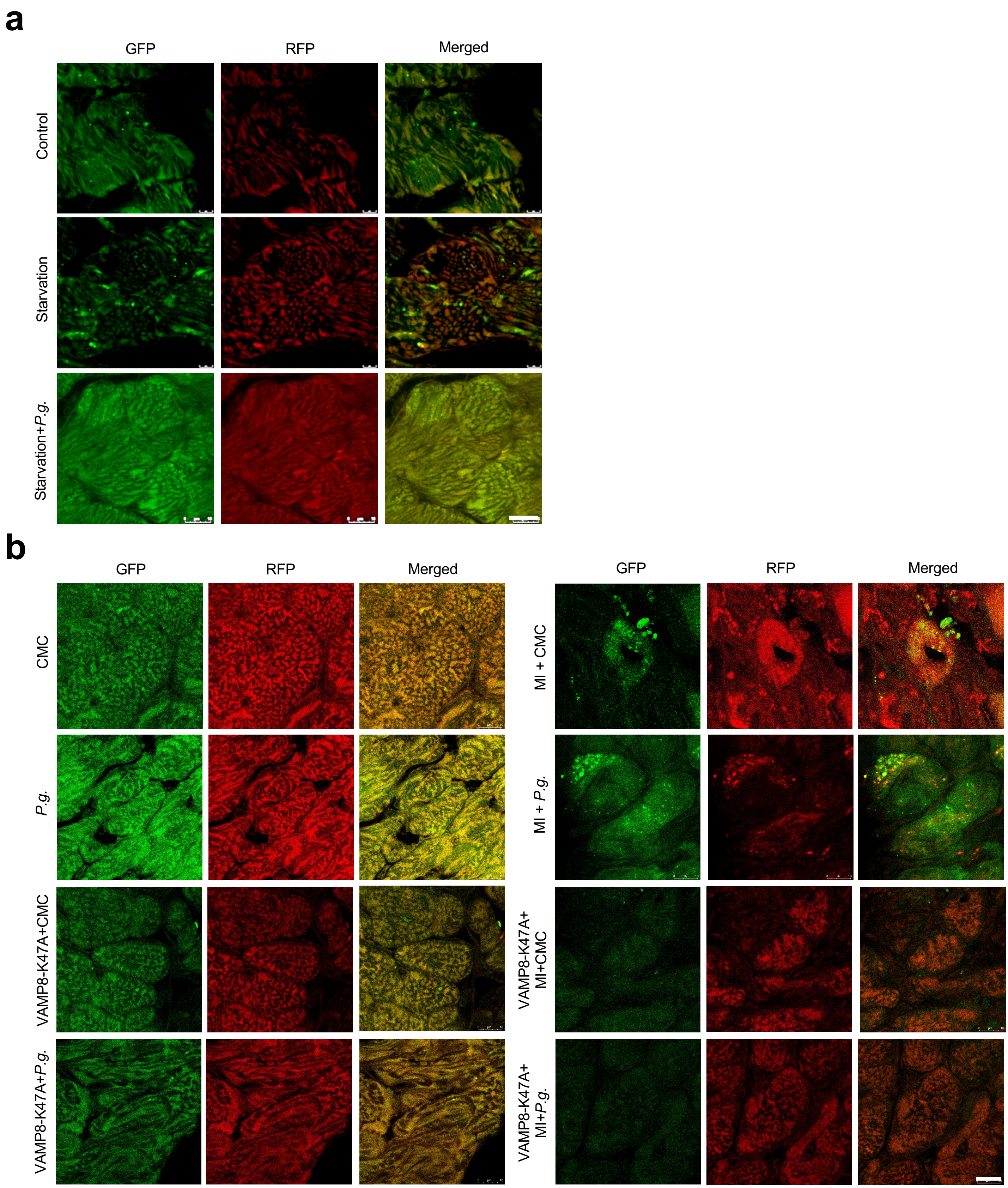

Fig. S6 a. Representative fluorescence ratio images of GFP-LC3-RFP-LC3ΔG transgenic mice, 48 h post starvation-GFP-LC3-RFP-LC3ΔG transgenic mice, and 48 h post starvation and WT*P.g.*-inoculated-GFP-LC3-RFP-LC3ΔG transgenic mice hearts. **b.** Representative fluorescence ratio images of CMC-inoculated mice, WT*P.g.*-inoculated mice, CMC-inoculated vesicle-associated membrane protein 8 (VAMP8)-K47A mice, WT*P.g.*-inoculated VAMP8-K47A mice, MI+CMC mice, MI+WT*P.g.* mice, VAMP8-K47A+MI+CMC mice, and VAMP8-K47A+MI+WT*P.g.* mice hearts.
